# Usefulness of an Automatic Quantitative Method for Measuring Regional Cerebral Blood Flow Using ^99m^Tc Ethyl Cysteinate Dimer Brain Uptake Ratio

**Published:** 2015

**Authors:** Rieko Nagaoka, Asato Ofuji, Kosuke Yamashita, Taeko Tomimatsu, Shinnichi Orita, Akihiro Takaki, Yoshikazu Uchiyama, Shigeki Ito

**Affiliations:** 1 Department of Radiology, Clinical Research Institute, National Hospital Organization Kyushu Medical Center, Fukuoka, Japan; 2 Graduate School of Health Sciences, Kumamoto University, Kumamoto, Japan; 3 Fujifilm RI Pharma Co., Ltd., Tokyo, Japan; 4 Department of Medical Imaging, Faculty of Life Sciences, Kumamoto University, Kumamoto, Japan

**Keywords:** ^99m^Tc-ECD, Brain uptake, Patlak plot, rCBF, SPECT

## Abstract

**Objective(s):**

Improved brain uptake ratio (IBUR), employing ^99m^Tc-ethyl cysteinate dimer (^99m^Tc-ECD), is an automatic non-invasive method for quantitatively measuring regional cerebral blood flow (rCBF). This method was developed by the reconstruction of the theory and linear regression equation, based on rCBF measurement by H_2_^15^O positron emission tomography. Clarification of differences in rCBF values obtained by Patlak plot (PP) and IBUR method is important for clinical diagnosis during the transition period between these methods. Our purpose in this study was to demonstrate the relationship between rCBF values obtained by IBUR and PP methods and to evaluate the clinical applicability of IBUR method.

**Methods:**

The mean CBF (mCBF) and rCBF values in 15 patients were obtained using the IBUR method and compared with PP method values.

**Results:**

Overall, mCBF and rCBF values, obtained using these independent techniques, were found to be correlated (*r*=0.68). The mCBF values obtained by the IBUR method ranged from 18.9 to 44.9 ml/100g/min, whereas those obtained by the PP method ranged from 34.7 to 48.1 ml/100g/min. The rCBF values obtained by the IBUR method ranged from 16.3 to 60.2 ml/100g/min, whereas those obtained by the PP method were within the range of 26.7-58.8 ml/100g/min.

**Conclusion:**

The ranges of mCBF and rCBF values, obtained by the IBUR method, were approximately 60% lower than those obtained by the PP method; therefore, this method can be useful for diagnosing lower flow area. Re-analysis of prior PP data, using the IBUR method, could be potentially useful for the clinical follow-up of rCBF.

## Introduction

Accurate quantification of regional cerebral blood flow (rCBF) is important for the clinical evaluation of cerebrovascular diseases, as well as other neurologic disorders (1). ^99m^Tc ethyl cysteinate dimers (^99m^Tc-ECD) have been widely used for the quantitative measurement of rCBF by single photon emission computed tomography (SPECT) (2-7).

Generally, accurate measurement of rCBF using SPECT requires arterial blood sampling by arterial puncture (2), which is an invasive and painful method for patients. Non-invasive quantitative measurements are useful for clinical studies since they employ simple and pain-free procedures without arterial blood sampling.

Several non-invasive quantitative measurement methods without arterial blood sampling have been proposed with the use of ^99m^Tc-ECD SPECT (4-7). Use of a new non-invasive quantitative measurement method, known as improved brain uptake ratio (IBUR), has been recently reported (8). This method changed the location of the region of interest (ROI) from the aortic arch to the ascending aorta (based on arterial blood flow dynamics) for obtaining an accurate input function (9-11). Furthermore, a regression equation for the IBUR method was constructed, based on H_2_^15^O positron emission tomography (H_2_^15^O PET), serving as the gold standard (8).

Both repeatability and reproducibility of the IBUR method were completely improved by the use of automatic ROI setting algorithm for determining the input function and 3-dimensional stereotaxic ROI template (3DSRT) algorithm for rCBF estimation (12, 13). For these reasons, the rCBF values obtained by the IBUR method can be evaluated in the same way as the rCBF values, obtained by H_2_^15^O PET.

The Patlak plot (PP) method, employing ^99m^Tc-ECD, has been widely used in clinical studies in Japan (3, 14). However, the repeatability and reproducibility of PP method are inferior to those of the IBUR, given the need for manual ROI setting and 2-dimensional imaging analysis. Thus, IBUR can replace PP method in clinical studies.

The accuracy of rCBF values obtained by PP method differs from that of IBUR, since the regression equation was constructed, based on the mean CBF (mCBF) measurement method that uses ^133^Xe (14).

For patient follow-up, clarifying the differences in rCBF values obtained by PP and IBUR methods is important for clinical diagnosis during the transition period between these two methods. Moreover, it is necessary to determine the optimal approach for evaluating the discrepancies in rCBF values between the PP and IBUR methods.

Our purpose in this study was to demonstrate the relationship between rCBF values, obtained by IBUR and PP methods and to evaluate the clinical applicability of IBUR method.

## Methods

Images were obtained from 15 patients (10 men, 5 women; age: 52–85 years; mean age: 71 years), undergoing both ^99m^Tc-ECD chest radio isotope (RI) angiography and SPECT examinations at the National Hospital Organization Kyushu Medical Center (Table 1).

**Table 1:** Patients’ characteristics in National Hospital Organization Kyushu Medical Center

Pt. No.	Age	Sex	Diagnosis
1	85	M	post CEA for asymptomatic stenosis of ICA,lt
2	84	M	Stenosis of both ICA
3	74	F	Aortic valve stenosis
4	59	F	mitral regurgitation
5	72	M	aortic regurgitation
6	73	M	Stenosis of ICA, lt
7	66	M	post CEA for asymptomatic stenosis of ICA,rt
8	77	M	post CEA for ICA stenosis,rt
9	62	M	post STA-MCA bypass for occlusion of ICA,rt
10	82	F	post STA-MCA bypass for occlusion of ICA,rt
11	69	F	Rheumatic aortic valve stenosis
12	75	M	Aortic valve stenosis
13	74	M	valvular disease of the heart
14	65	M	Stenosis of ICA,rt
15	52	F	Stenosis of ICA,rt
Ave.	71		

CEA: carotid endarterectomy

ICA: internal carotid artery

STA-MCA: superficial temporal artery to middle cerebral artery

lt.: leftrt.: right

None of the patients had pulmonary diseases. The studies were approved by the institutional ethics board of this institution, and written informed consents were obtained from all the patients or their next of kin.

### 

#### ^99m^Tc-ECD imaging

^99m^Tc-ECD imaging was performed at each of the facilities, using a dual-head SPECT scanner (E-cam, Siemens, Erlangen, Germany). ^99m^Tc-ECD RI angiographic images of the anterior brain and chest at a 15° left-anterior-oblique (LAO15) view were simultaneously obtained for 2 min (1 s/frame, 128×128 matrix), using a detector, equipped with low-energy high-resolution collimators and a 140 keV ± 7.5% energy window after a bolus injection of 600 MBq of ^99m^Tc-ECD; the pixel size was 4.00 mm.

The LAO15 position of the chest was obtained by posture modification. The head position was fixed at the anterior position at the time of imaging studies. After ^99m^Tc-ECD chest RI angiography, SPECT was performed with a 30-minute mid-scan time. The projection data were acquired every 150 sec by the continuous rotation of detectors by 180° (60 steps/180 degrees/150 sec, 128×128 matrix).

The SPECT images were obtained using 2-dimensional ordered-subset expectation maximization (2D-OSEM) method (subsets: 5, iterations: 20) (8). Scatter correction was not performed in order to apply the optimal 2D-OSEM condition, reported by Ito et al. (8). An attenuation coefficient of 0.09 cm^-1^ and a Butterworth pre-filter (cut-off: 0.5 cycle/cm, order: 8) were used for image reconstruction (8).

#### CBF analysis by PP method

A time-activity curve (TAC) for the arterial input function of RI angiographic images was obtained by setting circular ROIs with a diameter of 4 pixels on the ascending aorta as the input function and cerebral hemisphere ROIs on the anterior brain as output functions (3, 14). The location of the ROI was determined manually by its identification on all dynamic images.

The brain perfusion index (BPI) was obtained by the analysis of TACs of the aortic arch and the normal side of the brain. Finally, the mCBF was calculated using ^133^Xe regression equation (14). The rCBF values were obtained by the conversion of total counts in brain SPECT into mCBF, using Lassen’s correction (15).

The Syngo MI Applications VA46B Brain Patlak Proc. Program (Siemens, Erlangen, Germany) was used for the analysis of the data obtained by the PP method. The rCBF of each region was calculated by the conversion of the integrated SPECT count of the basal ganglia to mCBF.

#### CBF analysis by the IBUR method

A TAC for the arterial input function of dynamic images was obtained by setting the circular ROIs with a diameter of 4 pixels on the ascending aorta, based on blood flow dynamics (8). The location of the ROI was determined automatically by identifying the region with the maximum count in the ascending aorta among all dynamic images (13).

The second peak of the TAC was fitted with gamma function, since the first peak indicated the pulmonary artery or lung activity, overlapping with that of the ascending aorta. The input counts were obtained by integrating their gamma functions. The SPECT counts were converted, using Lassen’s correction (15).

The regional brain uptake ratio (rBUR) was obtained directly by dividing the SPECT counts of the same region by the input function and multiplying by cross calibration factor (CCF) between the planar image and SPECT image counts. Finally, the rCBF was calculated by applying H_2_^15^O PET regression equation (8). The process from ROI setting in dynamic images to the calculation of rCBF values was performed automatically with the original analysis software, using C++ (12).

The mCBF was calculated by averaging the rCBF values of the basal ganglia. The mCBF value was obtained, using the normal side in each patient.

#### PP vs. IBUR method

All SPECT images were analyzed using a 3DSRT on anatomically standardized CBF SPECT images for objective estimation of rCBF (13).

The 3DSRT is composed of 12 segments on each side: 1) anterior, 2) precentral, 3) central, 4) parietal, 5) angular, 6) temporal, 7) occipital, 8) pericallosal, 9) lenticular nucleus, 10) thalamus, 11) hippocampus, and 12) cerebellum.

The rCBF values were obtained, using 3DSRT. The mCBF and rCBF values, obtained by IBUR method, were compared with the values obtained by the PP method.

## Results

**Figure 1:**
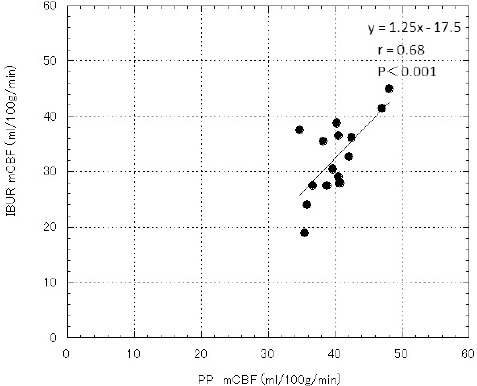
Correlation between mCBF values obtained by PP and IBUR methods; the regression equation for mCBF was expressed as y= 1.25x-17.5 (*r*=0.68, *P*<0.001)

Figure 1 shows the linear regression analyses for mCBF measurements by the PP and IBUR methods. Individual mCBF values, obtained by these independent techniques, were found to be correlated (*r*=0.68, *P*<0.001).

**Figure 2:**
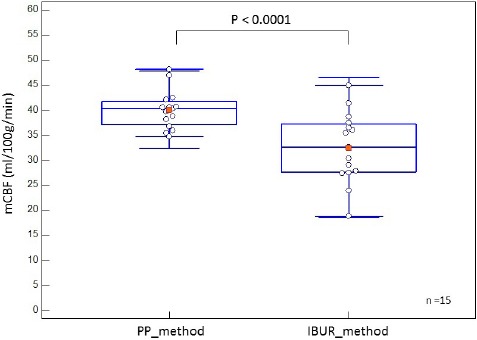
Box-and-whiskers plots comparing mCBF values of the PP and IBUR methods

Figure 2 shows box-and-whiskers plots comparing mCBF values obtained by PP and IBUR methods. The mCBF of PP method was significantly higher than that of the IBUR method (*P*<0.0001). The IBUR flow values were found to range between 18.9 and 44.9 ml/100g/min (median: 32.6, IQR: 27.6–37.3 ml/100g/min, and variance; 48.7), and the PP flow values were within the range of 34.7-48.1 ml/100g/min (median: 40.3, IQR: 37.1–41.8 ml/100g/min, and variance; 14.8) (Table 2). The values obtained by the IBUR method were approximately twice the PP values.

**Table 2:** Comparison of mCBF and rCBF values between the PP and IBUR methods

	PP_mCBF	IBUR_mCBF	PP/IBUR (mCBF)	PP_rCBF	IBUR_rCBF	PP/IBUR (rCBF)
Sample size	15	15		360	360	
Arithmetic mean	40.1	32.5		38.4	34.6	
Standard deviation	3.8	7.0		5.8	8.3	
Variance	14.8	48.7	0.3	33.0	62.7	0.5
Lowest value	34.7	18.9	1.8	26.7	16.3	1.6
Highest value	48.1	44.9	1.1	58.8	60.2	1.0
Median	40.3	32.6		37.3	33.6	
25th percentile	37.1	27.6		34.2	28.0	
75th percentile	41.8	37.3		42.0	39.8	
Two-tailed probability		*P* < 0.0001			*P* < 0.0001	

**Figure 3:**
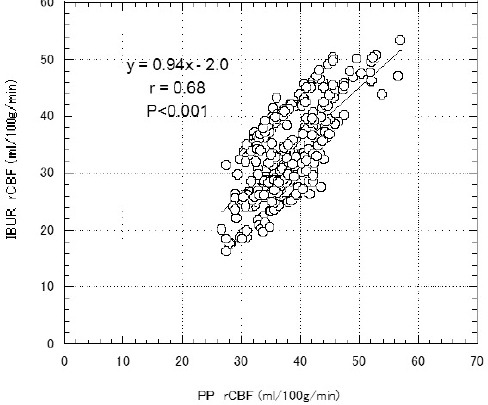
Correlation between PP and IBUR (rCBF values); the regression equation for rCBF was expressed as y = 0.94x - 2.0 (*r*=0.68, *P*<0.001)

Figure 3 shows the linear regression analyses for rCBF measurements with PP and IBUR methods. Individual rCBF values obtained using these independent techniques were found to be correlated (*r*=0.68, *P*<0.001).

**Figure 4:**
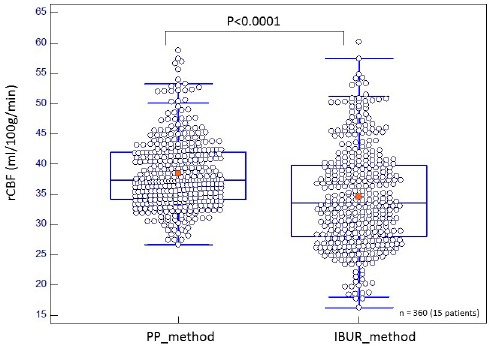
Box-and-whiskers plots comparing rCBF values of PP and IBUR methods

Figure 4 shows box-and-whiskers plots comparing rCBF values of the PP and IBUR methods. The rCBF of the PP method was significantly higher than that of the IBUR method (*P*<0.0001). The IBUR flow values were found to range from 18.7 to 60.2 ml/100g/min (median: 34.0, IQR: 28.5–40.1 ml/100g/min, variance: 62.7), and the PP flow values were within the range of 26.7-58.8 ml/100g/min (median: 37.4, IQR: 34.5–42.1 ml/100g/min, variance: 33.0) (Table 2). The highest rCBF value of the IBUR method was approximately equal to that of the PP method. However, the lowest rCBF value of the IBUR method was significantly lower than that of the PP method. The rCBF values of the IBUR method had an approximately 60% wider range of low blood flow.

## Discussion

We compared the mCBF and rCBF values, obtained by the IBUR method, with the corresponding values, obtained by the PP method in order to assess the applicability of IBUR for clinical studies.

In the IBUR method, rCBF is directly calculated using the rBUR method and regression equation (8). In the PP method, firstly, the mCBF is calculated by using BPI, and rCBF is converted using mCBF, according to the SPECT count distribution (3). The BPI and BUR values were not compared since the measurement process of the IBUR method is different from that of the PP method (3, 8).

A good linear correlation was not found between the BUR values obtained by the IBUR and PP methods (*r*=0.68) (Figure 1). The mCBF and rCBF values of the PP method were significantly higher than those of the IBUR method (Figures 2 & 4, Table 2). This is attributable to differences in the determination processes of input and output functions between the PP and IBUR methods. In the PP method, the input function is determined by setting the ROI on the aortic arch of the planar RI angiographic images, and the output function is determined by the ROI on the right–left brain RI angiographic images (3).

Thus, the ^99m^Tc aortic counts as an input function are underestimated by approximately 25% due to blood velocity changes, caused by collisions with the vascular wall and blood-flow loss due to vascular bifurcations (9); this causes fluctuations in the input function and mCBF in the PP method. Brain ^99m^Tc-ECD counts of the planar angiographic images as an output function are also underestimated due to gamma-ray absorption by bone and brain tissues. Therefore, these two underestimated values are offset by the BPI calculation process.

The range of values obtained by the IBUR method was approximately twice that of the PP method values (Figure 2 and Table 2). The rCBF values of the IBUR method had an approximately 60% wider range of low blood flow (Figure 4 and Table 1). These are due to differences in regression equations between the PP and IBUR methods. The regression equation of the PP method is expressed as:

mCBF (ml/100g/min)=2.60×(BPI)+19.8 (Eq 1)

Also, the regression equation for the IBUR method is expressed as:

rCBF (ml/100g/min)=3.23×(BUR)+4.66 (Eq 2)

The intercept of equation 1 is 19.8 ml/100g/min, corresponding to the minimum blood flow obtained in the PP method (3). In contrast, the lowest blood flow by the IBUR method is only 4.66 ml/100g/min (Eq 2) (2). Thus, the PP method is overestimated in a lower CBF region. For this reason, diagnosis of a low-blood-flow region for the PP method cannot but use relative evaluation with a high blood-flow region.

In contrast, taking into consideration that rCBF values of the IBUR method are equivalent to those of H_2_^15^ O-PET reported by Ito et al. (8), rCBF values of the IBUR method can be directly evaluated. Therefore, the IBUR method must be more useful than the PP method, and we anticipate that the IBUR method might replace the PP method in many clinical facilities.

Replacement of PP with IBUR method during the transition period presents a clinical challenge. When an ascending artery is detected on RI angiographic images, the optimal approach appears to be a re-analysis using the IBUR method, since it can be automatically completed in a few minutes (8, 12).

Generally, the RI angiographic images for the PP method were obtained at the anterior position; thus, the input function might have been underestimated due to absorption by sternum and loss of bifurcations in the blood flow (9).

Although these re-analyzed and converted values are approximations using the correction factor of the input function reported by Inoue et al. (9), they can be potentially useful for the follow-up of patients, requiring ongoing rCBF evaluations.

We observed that the rCBF values of the IBUR method have an approximately 60% wider range of low blood flow, compared to the PP method; therefore this method is useful for the diagnosis of low flow regions.

Taking into consideration that rCBF values of the IBUR method are equivalent to those of H_2_^15^O-PET, reported by Ito et al. (8), IBUR method is more useful than the PP method. However, these results are only related to one institution, using one equipment. Therefore, further verification is required in multiple facilities.

## Conclusion

The applicability of IBUR method in clinical studies was demonstrated by comparing CBF values, obtained by the IBUR and PP methods. The CBF values with IBUR method had a wider range for blood flow, compared to those of PP method. Therefore, the IBUR method may be more useful for the diagnosis of low flow area than the PP method. By comparing the previous data obtained by the PP method with those of the IBUR method, the applicability of IBUR method for patients requiring rCBF follow-up evaluations is demonstrated.
